# Concept of the term long lie: a scoping review

**DOI:** 10.1186/s11556-023-00326-3

**Published:** 2023-08-29

**Authors:** Jenny Kubitza, Iris T. Schneider, Bernd Reuschenbach

**Affiliations:** 1 Department of Psychosomatic Medicine and Psychotherapy, Spiritual Care and Psychosomatic Health, University Hospital Rechts Der Isar, Technical University of Munich, Kaulbachstraße 22a, Munich, 80539 Germany; 2 Department of Nursing Science I, University Trier, Trier, Germany; 3 Department of Nursing Science, University of Applied Science, Regensburg, Germany; 4 Department of Health and Care, Catholic University of Applied Sciences, Munich, Germany

**Keywords:** Long lie, Inability to get up, Lying in one position, Fall, Concept, Scoping review

## Abstract

**Background & aims:**

The term “long lie” is often used when individuals who have fallen are unable to stand up on their own, so they have to lie unintentionally for a longer period of time until they are noticed and can be helped. Although long lie can lead to both short- and long-term physical and psychological effects, little is known about what describes the term. The aim of this review is to identify what characterizes the term.

**Methods:**

Using the Arksey and O’Malley’s framework for scoping reviews in accordance with the modified *Preferred Reporting Items for Systematic Reviews and Meta-Analyses* framework, a systematic search was conducted for papers and gray literature that define, explain, or describe a long lie. The literature research was conducted via seven databases and Google Scholar.

**Findings:**

The search yielded 921 hits, of which 22 research papers are included; most studies were published after 2010. Emergency medicine and public health in particular have studied long lies and have found that it does not only affect the older adults who have fallen and cannot stand up on their own because of their frailty but also individuals with restricted mobility, which can be related to several reasons.

**Conclusions:**

The results show that a standard concept of a long lie is lacking. The duration of lying and the location alone are not relevant criteria. Further factors (helplessness, psychological and physical consequences, etc.) should also be taken into account.

**Supplementary Information:**

The online version contains supplementary material available at 10.1186/s11556-023-00326-3.

## Introduction

One in three older adults over 65 years of age fall once a year [1]. Although the risk of falling is high, the older adults are rarely aware of how they should act after a fall [2, 3], so they are often unable to escape the fall situation [4–6]. 78% of individuals aged 65 years and older who fall need help to get up from the floor [6]. As almost half of all falling incidents occur when the individuals are alone, they need to wait for a longer time until they are discovered and helped. On average, older adults wait nine to twenty minutes until they get help. In some cases, fallen individuals reported they had been on the floor for over one hour before they were able to contact the emergency service [6].

While the person is lying on the ground, serious physical and psychological effects can arise. Abnormal laboratory parameters, dehydration, rhabdomyolysis, sepsis, infections, pressures, and loss of consciousness have been reported [7–11]. In addition to acute and short-term consequences, community workers and psychologists have reported that people who have fallen are psychologically traumatized as a result of the predicament, which often leads to a strong fear of falling, limited mobility, becoming dependent, and losing their quality of life [3].

In this context, the term “long lie” is often used [2, 4, 6, 9–11]. At present, little is known about what exactly is meant by long lie and which attributes characterize the term. In some cases, a long lie is described as a consequence of a fall in which people remain on the floor for one hour or longer [2, 6, 12]. In other cases, the term is determined neither by a fall nor by a fixed time period [4, 9, 11]. There is also a reference to people who can no longer stand up but are able to move around the floor and contact help [10].

A preliminary search of the Cochrane Database of Systematic Reviews did not yield any current or in-progress reviews on the characteristics of a long lie. One review on the treatment of long lie was identified, but this focused mainly on the consequences of a long lie and the resulting therapy and prevention [13].

The current world guideline for falls prevention and management for older adults clearly identifies long lie for the first time, including the risks associated with it and how it can be prevented [14]. As more people are expected to be affected by a long lie in the future due to a significant demographic change and the increasing singularization of society in Western countries [15], it is important to clarify the concept of long lie.

## Methods

A scoping review was conducted to examine the term and to clarify the concept by scientific literature [16]. This scoping review followed to the PRISMA statement [16, 17] and the five-step framework by Arksey and O´Malley for scoping reviews [18]: (1) identifying the research question, (2) identifying relevant studies, (3) study selection, (4) charting the data, and (5) collating, summarizing, and reporting the results.

### Identifying the research question(s)

The following research questions guided this review:

- What are the characteristics of a long lie?
- What are the antecedents and consequences of a long lie?
- How is the quality of the studies on the term long lie?

### Identifying relevant literature

#### Search strategy

To identify the relevant documents, the following databases were searched from October to November 2022: MEDLINE (via PubMed), CINAHL, Cochrane Library, Science Direct, PLoS, GeroLit, and Scopus. To ensure that all relevant information was included, the gray literature and unpublished studies were searched using Google Scholar. Citation tracking was also performed on the included articles.

#### Search terms

The search strategy was developed by two authors (JK, IS) and was pilot-tested to pre-select keywords from abstracts and titles of papers considered relevant to the topic. The search strategy was a combination of the key concepts and search terms (Table 1), and was applied to all nine databases and adapted as needed.

**Table 1:** Key concepts and search terms

Key concepts	Search terms and synonyms	MeSH Terms PubMed
Population	long lie* OR inability to get up OR fall management	Falls, Accidental falls
Concept	definition* OR concept* OR character* OR indicator* OR identification* OR diagnos* OR descrip* OR explain*	Self Concept, Physiological Phenomena, Diagnosis
Context	As all setting, no keywords	

No time or language restrictions were used in the database search; for pragmatic reasons, only English- and German-language articles were included. The final search strategy is available in Additional file 1: Appendix 1.

### Selecting the literature

One reviewer (JK) combined and entered all database searches to EndNote reference management software (20.4/2022). After the removal of duplicates, the titles and abstracts have been screened against the eligibility criteria using the COVIDENCE platform. Sources were included that examined people who have suffered or treated a long lie, the inability to get up, or fall management. No further specific aspects were defined, as the characteristics of the term long lie were identified during the review. Included sources from the published research were qualitative and quantitative studies which define, describe, or explain a long lie. Discussion papers, letters, and websites are excluded. Books, book chapters, and other systematic/scoping reviews were also excluded, because this scoping review only used primary data.

### Charting the data

The authors used a data-extraction sheet to collect data on publication date, country, research question, study design, measures, discipline, context, sample size, characteristics of the sample, and description, causes, and consequences of a long lie. The quality of the study design was assessed using the Standard Quality Assessment Criteria [19], but no study was excluded based on quality. In case of uncertainties and disagreements in study selection, data extraction, and study quality, a consensus process has been followed. In these cases, all reviewers have compared and discussed their arguments until a decision was reached by common consent.

### Collating, summarizing and reporting the literature

The main findings were summarized and sorted to answer the research questions. The results are presented regardless of their methodological quality. Results were presented in a narrative format or in a visual representation.

## Findings

**Fig. 1:**
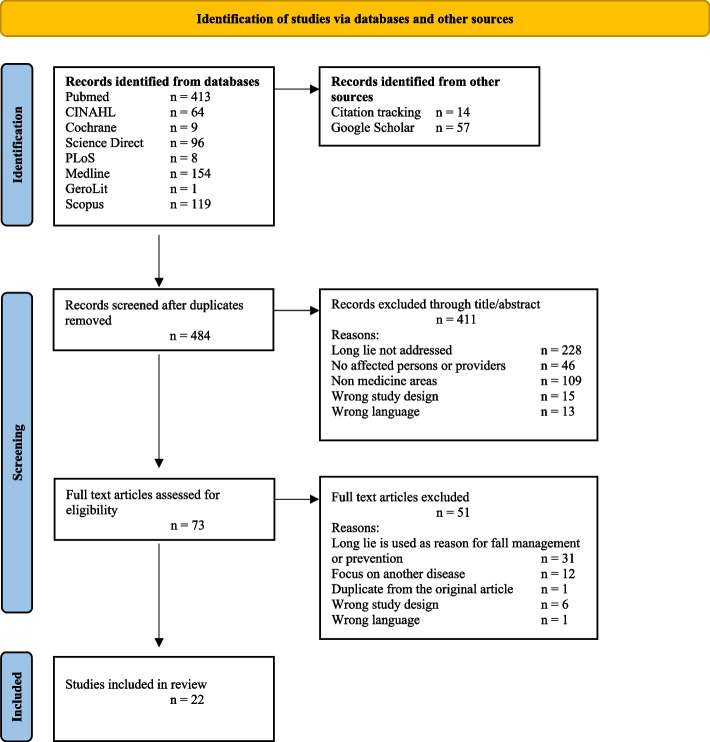
PRISMA flow diagram

The search yielded 921 records. Using citation tracking, 14 additional publications were added to the search. Duplicates were removed and the remaining titles and abstracts were screened against the defined criteria, leaving 73 records for full-text review. 51 articles that did not meet the inclusion criteria were excluded during the full-text review, so the review includes a total of 22 studies. The reasons for study exclusion are explained in the PRISMA flow diagram (Fig. 1).

### Study characteristic

The 22 studies have been published between 1981 [20] and 2022 [21, 22], whereby most studies were published after 2010 (*n* = 11). Prior to 2010, studies were conducted only in Finland (*n* = 1), France (*n* = 1), the UK (*n* = 4), and the USA (*n* = 5), the newer studies were done in Australia (*n* = 1), Germany (*n* = 7), Switzerland (*n* = 1), UK (*n* = 1), and USA (*n* = 1). Most of the studies (*n* = 15) were conducted with a quantitative design. Studies with qualitative design (*n* = 3) and case reports (*n* = 4) were carried out in Germany, Switzerland, and the USA. A total of 6311 individuals have been included in the studies, although not all persons were affected by a long lie. 2805 people were unable to get up from the floor and 1121 people were affected by a long lie, whereby the researchers had different definitions and thresholds of a long lie, so it cannot be assumed that the people spent the same amount of time on the ground (Table 2). Of the seven studies conducted in Germany, a rather small number of 102 participants were examined, whereby the study from Schwickert et al. [23] is not included, because they only reported falls and it is not apparent how many people were involved in the falls (*n* = 34 falls with delayed recovery). The researchers of the study conducted in Switzerland interviewed experts on the treatment of long lies [24].

**Table 2:** Summary of relevant studies (*N* = 22)

Authors (Year)	Country	Research question	Study Design	Measures	Discipline and context	Sample	Definition of the term long lie
**Adams & Tyson (2000)** [25]	UK	How effective is the backward chaining approach in teaching and enabling an older adult with a history of falls to get up from the floor?	Quantitative	Case description with a two-standard deviation band method	Physiotherapy Outpatient	Person affected by a long lie (14 h) *n* = 1	Persons are unable to get up from the floor on their own
**Baraff et al. (1999)** [26]	USA	How does a practice guideline affect the care of older adults who have fallen in the emergency department?	Quantitative	Pre-post intervention study	Emergency medicine Inpatient	Person affected by a long lie (≥ 5 min) pre *n* = 143 post *n* = 11	Persons are unable to get up from the floor on their own and lie for more than 5 min
**Bisson et al. (2015)** [27]	USA	What is the prevalence of initial recovery and long lie in fallen persons with multiple sclerosis?	Quantitative	Secondary analysis from a cross-sectional descriptive study	Rehabilitation medicine Outpatient	Persons affected by a delayed initial recovery *n* = 89 Persons affected by a long lie (≥ 1 h) *n* = 15	Persons are remaining on the floor or ground for more than 1 h
**Erhard, D. (2022)** [22]	GER	How are older adults treated if they have not been seen for several days?	Unclear	Case description	Emergency medicine Outpatient	Persons affected by a long lie (4d) *n* = 2	Persons are remaining on the floor or ground for a longer time
**Fleming et al. (2008)** [28]	UK	What is the prevalence of lying on the floor for a long time in fallen and the oldest old adults?	Quantitative	Prospective cohort study	Nursing Outpatient	Persons affected by lying on the floor (< 1 h) *n* = 85 Persons affected by a long lie (≥ 1 h) *n* = 20	Persons are unable to get up from the floor on their own and lie for more than 1 h
**Fischer (2019)** [24]	CH	What are the characteristics of a long lie?	Qualitative	Interviews	Emergency medicine Inpatient	Experts in the treatment of long lies *n* = 4	Special form of trauma in which persons are unable to get up and lie on hard ground for a longer period
**Gräff et al. (2018)** [29]	GER	How are patients treated with social breakdown?	Quantitative	Monocentric retrospective observation study	Emergency medicine Inpatient	Persons affected by a long lie (≥ 1d) *n* = 17	Persons are unable to move on their own; this could affect the upper and lower extremities
**Gurley et al. (1996)** [30]	USA	What is the prevalence of older adults found helpless or dead in their homes and what is the impact on their health?	Quantitative	Prospective cohort study	Public health Inpatient and outpatient	Persons affected by a long lie and found alive *n* = 297 Persons affected by a long lie and found dead *n* = 90	No definition
**Häcker & Offterdinger (2019)** [31]	GER	How is a long lie related to a cardiac arrest?	Unclear	Case description	Emergency medicine Inpatient	Person affected by a long lie (5d) *n* = 1	Persons are unable to move on their own and lie in a helpless position for a longer period
**Hayes et al. (2003)** [32]	USA	How are older adults treated with a hip fracture?	Unclear	Case description	Rehabilitation Medicine Inpatient	Person affected by a long lie (20 h) *n* = 1	Persons are unable to move on their own and lie on the floor for a longer period
**Hierholzer et al. (2011)** [33]	GER	What complications can be expected with a long lie?	Unclear	Case description	Emergency medicine Inpatient	Person affected by a long lie (1d) *n* = 1	Persons are unable to move on their own and lie on hard ground for a longer period
**Hüser et al** **(2022)** [21]	GER	Which factors influence morbidity and mortality in patients with a long lie?	Quantitative	Monocentric retrospective observation study	Emergency medicine Inpatient	Persons affected by a long lie (≥ 7 h) *n* = 50	People are unable to get help
**Kubitza & Reuschenbach (2021)** [34]	GER	What are the characteristics of a long lie?	Qualitative	Grounded theory and conceptual analysis with narrative interviews	Nursing Inpatient and outpatient	Persons affected by a long lie (≥ 1 h) *n* = 4	Suddenly restricted mobility in an unwanted situation of lying or sitting on a deeper level for several hours to days
**Reece & Simpson (1996)** [35]	UK	How do the backward and forward chaining approaches differ in terms of acceptance and learning success for older adults who have fallen?	Quantitative	Descriptive experimental study	Physiotherapy Inpatient	Persons affected by lying on the floor *n* = 18	Persons are unable to get up from the floor on their own and lie for a longer period
**Ryyänen et al. (1992)** [36]	FI	What are the characteristics of older adults who fall and lay in position for 15 min or more?	Quantitative	Case control study	Public Health Inpatient and outpatient	Persons affected by lying on the floor (< 15 min) *n* = 57 Persons affected by lying on the floor (< 1 h) *n* = 33 Persons affected by lying on the floor (≥ 1 h) *n* = 33	People lie for more than 15 min
**Schwickert et al. (2018)** [23]	GER	How do self-recovered falls differ from non-recovered falls with long lies?	Quantitative	Prospective observation study	Geriatric medicine Inpatient and outpatient	Persons affected by a fall *n* = 27	Persons are remaining on the floor or ground for more than 10 min
**Scott (2020)** [37]	UK	What are the characteristics of older service users who have fallen and been referred to a falls prevention service by paramedics?	Quantitative	Retrospective cross-sectional cohort study	Public health Outpatient	Persons affected by a long lie (≥ 1 h) *n* = 23	Persons are remaining on the floor or ground for more than 1 h
**Simpson et al. (2014)** [38]	AUS	What are the characteristics of older adults who fall and call an emergency ambulance?	Quantitative	Prospective cohort study	Public health Outpatient	Persons affected by lying on the floor (< 1 h) *n* = 849 Persons affected by a long lie (≥ 1 h) *n* = 202	Persons are remaining on the floor or ground for more than 1 h
**Tinetti et al. (1993)** [39]	USA	What are the characteristics and prognosis of older adults with inability to get up after falling?	Quantitative	Longitudinal prospective cohort study	Public health Inpatient and outpatient	Persons affected by lying on the floor *n* = 148	Persons are unable to get up from the floor on their own
**Tischler & Hobsen (2005)** [40]	USA	Which consequences do older adults fear if they fall?	Qualitative	Semi-structured interviews	Rehabilitation medicine Outpatient	Persons with fear of falling *n* = 7	Persons are unable to get up from the floor on their own or get help in a reasonable time
**Vellas et al** **(1987)** [41]	FR	How does a fall affect activity in older adults?	Quantitative	Prospective case control study	Public health Outpatient	Persons affected by lying on the floor (< 1 h) *n* = 71 Persons affected by a long lie (≥ 1 h) *n* = 6	No definition
**Wild et al** **(1981)** [20]	UK	How dangerous are falls in older adults at home?	Quantitative	Longitudinal prospective cohort study	Geriatric medicine Outpatient	Persons affected by a long lie (≥ 1 h) *n* = 20	Persons are remaining on the floor or ground for more than 1 h

### Study quality

Two independent reviewers have conducted the study quality appraisal following the Standard Quality Assessment Criteria by Kmet et al. [20]. The score sheet for qualitative studies consists of ten items, and the one for quantitative studies has fourteen items. As each investigator could rate the items with a maximum of two points, the single items could score with a maximum of four points (2 reviewers × 2 points).

**Fig. 2:**
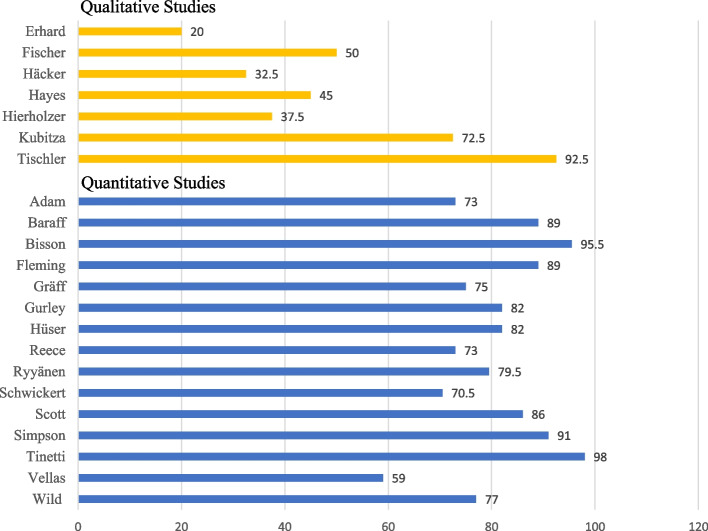
Quality assessments results for qualitative and quantitative studies according to the Standard Quality Assessment Criteria [19]. The qualitative studies could reach a maximum score of 40 points (2 reviewers × 10 items × 2 points), the quantitative studies of 44 points (2 reviewers × 14 items × 2 points – (2 reviewers × 3 items × 2 points)). The results are presented as a percentage of the maximum scores

The quantitative studies rate better with a mean score of 81.3% than the qualitative studies with a mean score of 50% (Fig. 2). The qualitative studies are rated lower because the four case descriptions in particular only described their methodological procedure inadequately, which means that the item data collection is rated 0.9 and the item´s data analysis and verification of data is 0.7 each. The quantitative studies are limited especially because the sample size is not appropriate (2.6) and there is a missing variance in the reporting of results (2.6). Furthermore, all quantitative studies do not use randomizing or blinding interventions (an overview of all items can be found in Additional file 1: Appendix 2 and 3).

### Use of the term long lie

The term “long lie” is most frequently used in the context of emergency care in seven studies [21, 22, 24, 26, 29, 31, 33]. The studies primarily focused on the acute treatment of a long lie, for which they surveyed the characteristic attributes of the incidence. The characteristics of long lie were also studied in the context of nursing [34], geriatrics [23], and public health [36, 39]. In addition to the characteristics, the prevalence of long lie was surveyed. This subject was pointed out in studies conducted by authors in the disciplines of nursing [28], rehabilitative medicine [27], and public health [30]. Often, long lies have been measured as a secondary outcome to identify the consequences of a fall [20, 40, 41]. Furthermore, authors in studies in public health examined the ambulance service [37, 38], and researchers in psychotherapy focused on two studies the stand-up strategies of persons in the context of fall management [25, 35].

In total, authors in two studies in the field of nursing [28, 34], two studies in the discipline of geriatrics [20, 23], three studies in rehabilitative medicine [27, 32, 40], and six studies in public health [30, 36–39, 41] described a long lie.

### Characteristics of the term long lie

#### Causes and associated factors

The risk of suffering a long lie increases significantly with age [25–30, 38–40]. Older adults who are affected are more often women [21, 27, 30, 35, 37], persons younger than 65 are mostly men [21, 29, 30], usually socially isolated [29, 31] and more often found dead [30, 31].

Older adults suffered falls [19–21, 23–28, 30, 32–41], but it has been the frailty of the individuals that affected their inability to get up after the fall [22–28, 30, 33–36, 39]. Older adults are characterized mainly by balance and mobility problems [27, 28, 36, 39], they have generally a longer disease duration with poor health [27, 29], and are dependent on others for help with activities of daily living [39]. Two studies have also indicated that the affected persons often suffer from a cognitive or mental illness [28, 29]. Schwickert et al. found that persons who take longer than 25 s to recover after a fall are often unable to get up by themselves [23].

Besides the fall, a long lie can also be initiated by immobilization caused by serious acute or chronic injuries [21, 24, 29, 30, 36, 39], neurological events with loss of consciousness [21, 24], infection with increased body temperature [21, 36], domestic trauma [21], low serum potassium concentration [36], intoxication [21, 24, 30], or suicide attempts [30].

In addition to the biological and social causes, the duration of the long lie depends on whether the persons have active social relationships [29–31] and how much time a day they spend alone in their own rooms [30, 39].

#### Consequences

Authors in three studies reported in detail which acute biological consequences occur after a long lie [21, 29, 30]. These symptoms are confirmed by further studies. Hypothermia [21, 22, 24, 29, 31], infections [21, 24, 29, 30, 39], exsiccosis [21, 22, 24, 29, 30, 32, 39, 41], wounds and serious injuries [22, 24, 28–30, 32, 39, 41], as well as pathological laboratory parameters [21, 29, 32, 33] up to rhabdomyolysis [21, 24, 31–33], and sepsis [21, 24, 29, 30] are the most common.

It becomes apparent that the length of immobilization influences the symptoms. While in Gräff et al. (2018) [29] and Hüser et al. (2022) [21] with mean lying times of 1.9 days and 18.8 h, the effects on the body occur in almost all persons, these already decrease with an average lying time of two hours [30]. This is also reflected in the mean hospitalization period, which is still 27 and 14 days for Gräff et al. (2018) [29] and Hüser et al. (2022) [21], and only two days for Gurley et al. (1996) [30].

The long lie also has a long-term effect on the bio-psycho-social domains, so that persons who cannot get up after a fall are more afraid of falling again (fear to fall) [25, 34, 40], limit their activities of daily living [33, 39, 41], and recontact the ambulance services more often (78.8% with long lie vs. 60.6% without long lie) [37]. Within one year, 29% [28] to 77% [30] of the persons move to a long-term care facility and mortality increases compared to persons who fell without a long lie [20, 39] (Table 3).

**Table 3:** Causes and consequences of long lie according to bio-psycho-social domains

Domain	Causes and associated factors	Consequences
Biological	Fall with frailty [21–23, 27–29, 31, 33–36, 38, 39] Acute or chronic injuries and trauma [20, 22, 24, 27, 34, 39] Intoxication [20, 22, 27]	Hypothermia, Infections, Exsiccosis, Injuries, Pathological laboratory parameters [20–22, 24–27, 29, 34, 36, 40] Restricted mobility [31, 34, 40]
Psychological	Suicide attempts [27]	Fear of falling [30, 31, 35]
Social	Alone most of the day [27, 34] Inactive social relationships to social withdrawal [24, 26, 27]	Dependence on others [31, 34, 40] Move to a long-term care facility [27, 36]

#### Indicators and definition

All researchers, except for two studies [30, 41], explained what they understand of long lies in their study. In the 20 definitions, one common characteristic was apparent: all researchers mentioned that the affected individuals are limited in their mobility. While Kubitza and Reuschenbach (2021) [34] wrote of limited mobility, the other researchers described the mobility restrictions. In most of the studies, the affected persons were unable to get up (*n* = 7) [24–26, 28, 35, 39, 40] or they remained in the same place (*n* = 7) [20, 22, 23, 27, 36–38]. In three studies, the affected persons were completely unable to move [29, 31, 32], while in two other studies, the persons were only unable to get help [21, 40]. Nine studies also added that the persons had to wait for help while lying [24, 26, 28, 31–36] or sitting [34].

Researchers from 13 studies described that affected people had limited mobility on the floor or ground [20, 22, 23, 25–28, 32, 35, 37–40]. Only studies from Germany or Finland did not specify the location explicitly and did not include the location of the event in the definition at all [21, 29, 31, 36], or alternatively used the terms hard ground [24, 33] or deeper level [34]. It becomes apparent that studies that were more explicit about location often included the fall in their research question [20, 23, 25–28, 35, 37–40].

In eight studies, a specific time was also used to determine long lie, ranging from five minutes [26], ten minutes [23], and fifteen minutes [36] to one hour or more [20, 27, 28, 37, 38]. While seven studies were more unspecific in this aspect and used the terms longer time [22, 24, 31–33, 35] or several hours to days [34], five studies did not use time restrictions at all [21, 25, 29, 39, 40].

## Discussion

Although the literature on long lies has increased significantly since 2010, concepts still vary. Either the studies use explanations from earlier research [12, 21, 27, 34, 37] or they use their own concepts, whereby these are not scientifically confirmed [22, 24, 29, 31, 33, 38]. Only one study from Germany developed a definition with a concept analysis, although the definition was collected with a small number of subjects [34]. However, none of the concepts are valid in general, so the results of the studies are still only comparable to a limited extent.

The studies so far mainly examined patients with a long lying time. On the one hand, this is due to the fact that milder cases often remain unnoticed [38], and on the other, in several studies, a long lie was just recorded as such after a lying time of at least one hour [2, 6, 20, 27, 28, 37, 38]. However, most studies refuse to terminate the long lie with a fixed time [9–11, 21, 22, 24, 25, 29, 31–35, 39, 40]. This makes sense for two reasons: (1) Lying time is measured either by self-report or by last-seen wells, which for various reasons leads to over- or underestimated immobility time and a high level of misreporting [20–22, 26–30, 32, 34–36, 38, 39, 41]; (2) The consequences significantly improve with increasing lying time, but they can already occur in less than one hour [27, 28, 30, 34–36, 38, 39, 41]. In fact, it is critical to examine whether every fall that requires the intervention of another person should not be considered as a potential long lie and therefore the lying time should be irrelevant. So it seems appropriate to focus more on the fact that the fall cannot be recovered and is experienced alone. For example, older persons living alone cannot expect to get help in an appropriate time after a fall, whereas persons in long-term care facility can trust to be found within minutes to a few hours; this difference affects the bio-psycho-social outcome.

This appreciation is already reflected in some definitions, who describe a long lie more as an event in which persons are unable to get up from the floor or other lower level on their own [21, 29, 32, 39].

Due to the missing common concept of long lie, there is the problem that the different definitions have an impact on the characteristics; e. g. if the persons are remaining on the floor or ground for at least one hour, this also affects the consequences [21, 29, 30]. It was also noted that studies examining people over the age of 65 often identified falls as the cause of long lie [20, 25, 26, 28, 32–41], while studies including people younger than 65 also identified that other factors can be responsible for a long lie [21, 24, 29, 30].

Consequently, a long lie should not be limited by the fact that it is experienced on the ground, is caused by a fall, or that the event must for at least one hour, because only then is it possible to holistically identify the characteristics of long lie. More recent studies already examine long lie independent of the factors of age, cause, and/or lying time [21, 24, 29, 31, 33, 34], but it now needs studies with a larger number of cases in a prospective and longitudinal design. In addition, there is an increasing demand to understand how people experience long lies psychologically and social, as previous studies have mainly focused on the acute and long-term physical consequences [7–11, 20–22, 24, 26–31, 33, 38, 39, 41]. This requires qualitative studies that are methodologically credible and explicitly ask about the psychological and social aspects of the experience.

For the first time, this scoping review has examined several studies on long lies to identify their characteristics with the aim of clarifying a long lie as a concept. Although a rigorous search strategy has been used, it is possible that not all relevant records have been identified.

## Conclusions

The relevance of long lies is increasing, both in its prevalence and in its consequences, which often means that the affected individuals become immobile and dependent on others. A long lie must be recognized as a serious incidence, and therefore needs a common definition that includes all affected persons, even those who do not fall and have to wait for help for less than one hour. A common definition has the further capability to extend the guidelines for fall prevention, to create policies and the use of health technology assessments.

### Supplementary Information

**Additional file 1:** **Appendix 1.** Search string for MEDLINE. **Appendix 2.** Quality assessment of single items in reporting qualitative data. **Appendix 3.** Quality assessment of single items in reporting quantitative data.

## Data Availability

The datasets used and/or analyzed during the current study available from the corresponding author on reasonable request.

## References

[1] Saß AC, Varnaccia G, Rommel A (2019). Sturzunfälle bei Erwachsenen. Ergebnisse der Befragung Gesundheit in Deutschland aktuell. Präv Gesundheitsf.

[2] Charlton K, Murray CM, Kumar S (2017). Perspectives of older people about contingency planning for falls in the community: a qualitative meta-synthesis. PLoS ONE.

[3] Charlton K, Murray CM, Kumar S (2016). Getting help quickly: older people and community worker perspectives of contingency planning for falls management. Disabil Rehabil.

[4] Johnston K, Worley A, Grimmer-Somers K, Sutherland M, Amos L (2010). Perspectives on use of personal alarms by older fallers. Int J Gen Med.

[5] Bergland A, Laake K (2005). Concurrent and predictive validity of “getting up from lying on the floor”. Aging Clin Exp Res.

[6] Johnston K, Worley A, Grimmer-Somers K, Sutherland M, Amos L (2010). Personal alarm use to call the ambulance after a fall in older people: characteristics of clients and falls. JEPHC.

[7] Marcus EL, Rudensky B, Sonnenblick M (1992). Occult elevation of CK as a manifestation of rhabdomyolysis in the elderly. J Am Geriatr Soc.

[8] Mallinson WJW, Green MF (1985). Covert muscle injury in aged patients admitted to hospital following falls. Age Aging.

[9] von Cranach M, Niesen W (2019). Aphasie und Vigilanzminderung – Fremdanamnese als Schlüssel zur Diagnose. DG Neurologie.

[10] Valek R, Von der Mark J (2017). Wenn der Patient sauer aber nicht süß ist. Med Klin Intensivmed Notfmed.

[11] Gleich J, Fürmetz J, Kamla C, Pedersen V, Böcker W, Keppler AM (2020). Gluteales Kompartmentsyndrom nach Liegetrauma bei Opiatabusus. Unfallchirug.

[12] Nyman SR, Victor CR (2014). Use of personal call alarms among community-dwelling older people. Ageing Soc.

[13] Kubitza J, Haas M, Keppeler L, Reuschenbach B (2022). Therapy options for those affected by a long lie after a fall: a scoping review. BMC Geriatr.

[14] Montero-Odasso M, van der Velde N, Martin FC, Petrovic M, Tan MP, Ryg J, Aguilar-Navarro S (2022). World guidelines for falls prevention and management for older adults: a global initative. Age Ageing.

[15] Schwickert L, Oberle C, Becker C, Lindermann U, Klenk J, Schwenk M, Boruke A, Ziljstra W (2016). Model development to study strategies of younger and older adults getting up from the floor. Aging Clin Exp Res.

[16] von Elm E, Schreiber G, Haupt CC (2019). Methodische Anleitung für Scoping Reviews (JBI-Methodologie). Z Evid Fortbild Qual Gesundhwesen.

[17] Tricco AC, Lilie E, Zarin W, O’Brien KK, Colquhoun H, Levac D, Moher D (2018). PRISMA Extension for scoping reviews (PRISMAScR): checklist and explanation. Ann Intern Med.

[18] Arksey H, O’Malley L (2005). Scoping studies: towards a methodological framework. Int J Soc Res Methodol.

[19] Kmet LM, Lee RC, Cook LS. Standard quality assessment criteria for evaluating primary research papers from a variety of fields*.* 2004. 10.7939/R37M04F16.

[20] Wild D, Nayak USL, Isaacs B (1981). How dangerous are falls in old people at home. Br Med J.

[21] Hüser C, Hackl M, Suarez V, Gräff I, Bernhard M, Brust V, Adler C (2022). Liegetrauma: retrospektive Analyse einer Patientenkohorte aus einer universitären Notaufnahme. Med Klin Intensivmed Notfmed.

[22] Erhard D (2022). Am Boden. Retten!.

[23] Schwickert L, Klenk J, Zijlstra W, Forst-Gill M, Sczuka K, Helbostad JL, Chiari L, Aminian K, Todd C, Becker C (2018). Reading from the black box: what sensors tell us about resting and recovery after real-world falls. Gerontology.

[24] Fischer M. Das Liegetrauma. Die höchste Form von Hilflosigkeit*.* 2019. https://www.notfallpflege.ch/files/_Demo/Dokumente/Das_Liegetrauma_Diplomarbeit_Finalversion.pdf. Accessed 15 Dec 2022

[25] Adams JMG, Tyson S (2000). The effectiveness of physiotherapy for enable an elderly person to get up from the floor: a single case study. Physiotherapy.

[26] Baraff LJ, Lee TJ, Kader S, Della PR (1999). Effect of a practice guideline on the process of emergency department care of falls in elder patients. Acad Emerg Med.

[27] Bisson EJ, Peterson EW, Finlayson M (2015). Delayed initial recovery and long lie after a fall among middle-aged and older people with multiple sclerosis. Arch Phys Med Rehabil.

[28] Fleming J, Brayne C (2008). Inability to get up after falling, subsequent time on floor, and summoning help: prospective cohort study in people over 90. BMJ.

[29] Gräff I, Dolscheid-Pommerich RC, Ghamari S, Goost H (2018). Verwahrlost, einsam und krank – der soziale Breakdown. Med Klin Intensivmed Notfmed.

[30] Gurley RJ, Lum N, Sande M, Lo B, Katz MH (1996). Persons found in their homes helpless or dead. J Am Geriatr Soc.

[31] Häcker A, Offterdinger M (2019). Liegetrauma und Bergungstod. Brandhilfe.

[32] Hayes KS, Steinke EE, Heilman A (2003). Case study of hip fracture in an older person. J Am Acad Nurse Pract.

[33] Hierholzer D, Eschbacher E, Wiesmann L, Busch HJ (2012). Liegetrauma eines 79-Jährigen. Akutes Nierenversagen als ernste Komplikation der Rhabdomyolyse. Rettungsdienst.

[34] Kubitza J, Reuschenbach B (2021). Gestürzt und über Tage hilflos allein. Pflege Z.

[35] Reece AC, Simpson JM (1996). Preparing older people to cope after a fall. Physiotherapy.

[36] Ryynänen OP, Kivelä SL, Honkanen R, Laippala P (1992). Falls and lying helpless in the elderly. Z Gerontol.

[37] Scott J (2020). Re-contact rates with a UK ambulance service following paramedic referral to a falls prevention service for those aged ≥ 65 years: a retrospective cohort study. Br Paramed J.

[38] Simpson PM, Bendall JC, Tiedemann A, Lord SR, Close JCT (2014). Epidemiology of emergency medical service responses to older people who have fallen: a prospective cohort study. Prehosp Emerg Care.

[39] Tinetti ME, Liu WL, Claus EB (1993). Predictors and prognosis of inability to get up after falls among elderly persons. JAMA.

[40] Tischler L, Hobson S (2005). Fear of falling: a qualitative study among community-dwelling older adults. Phys Occup Ther Geriatr.

[41] Vellas B, Cayla F, Bocquet H, de Pemille F, Albarede JL (1987). Prospective study of restriction of activity in old people after falls. Age Aging.

