# Supplementary material for: Concept of the term long lie: a scoping review
Source: Eur Rev Aging Phys Act. 2023 Aug 29;20:16. doi: 10.1186/s11556-023-00326-3 (PMC10463813; doi:10.1186/s11556-023-00326-3)
Supplement: Supplementary file 1 — Additional file 1: Appendix 1. Search string for MEDLINE. Appendix 2. Quality assessment of single items in reporting qualitative data. Appendix 3. Quality assessment of single items in reporting quantitative data. [file 11556_2023_326_MOESM1_ESM.docx]

Appendix 1: Search string for MEDLINE

| **Search string for MEDLINE via PubMed** |
| --- |
| (long lie* OR  inability to get up OR  fall management OR  Falls [MH] OR  Accidental falls [MH])  AND  (definition* OR  concept* OR  character* OR  indicator* OR  identification* OR  diagnos* OR  descrip* OR  explain* OR  Self Concept [MH] OR  Physiological Phenomena [MH] OR  Diagnosis [MH]) |

Appendix 2: Quality assessment of single items in reporting qualitative data

*Quality assessment of single items in reporting qualitative data according to the Standard Quality Assessment Criteria (Kmet et al., 2004). To reflect on the overall study reporting quality, the reporting quality of the single item across the included studies is considered. As each investigator could rate the items with a maximum of 2 points, the single items could score with a maximum of 4 points (2 reviewers x 2 points).*

Appendix 3: Quality assessment of single items in reporting quantitative data

*Quality assessment of single items in reporting quantitative data according to the Standard Quality Assessment Criteria (Kmet et al., 2004). To reflect on the overall study reporting quality, the reporting quality of the single item across the included studies is considered. As each investigator could rate the items with a maximum of 2 points, the single items could score with a maximum of 4 points (2 reviewers x 2 points).*
